# Effect of Sound Coding Strategies on Music Perception with a Cochlear Implant

**DOI:** 10.3390/jcm11154425

**Published:** 2022-07-29

**Authors:** Gaëlle Leterme, Caroline Guigou, Geoffrey Guenser, Emmanuel Bigand, Alexis Bozorg Grayeli

**Affiliations:** 1 Otolaryngology, Head and Neck Surgery Department, Dijon University Hospital, 21000 Dijon, France; gaelle.leterme@chu-reunion.fr (G.L.); gg@histoiredentendre.fr (G.G.); alexis.bozorggrayeli@chu-dijon.fr (A.B.G.); 2 ImVia Research Laboratory, Bourgogne-Franche-Comté University, 21000 Dijon, France; 3 LEAD Research Laboratory, CNRS UMR 5022, Bourgogne-Franche-Comté University, 21000 Dijon, France; emmanuel.bigand@u-bourgogne.fr

**Keywords:** music perception, hearing function, cochlear implant, sound processing strategy, pitch perception, rhythm perception

## Abstract

The goal of this study was to evaluate the music perception of cochlear implantees with two different sound processing strategies. Methods: Twenty-one patients with unilateral or bilateral cochlear implants (Oticon Medical^®^) were included. A music trial evaluated emotions (sad versus happy based on tempo and/or minor versus major modes) with three tests of increasing difficulty. This was followed by a test evaluating the perception of musical dissonances (marked out of 10). A novel sound processing strategy reducing spectral distortions (CrystalisXDP, Oticon Medical) was compared to the standard strategy (main peak interleaved sampling). Each strategy was used one week before the music trial. Results: Total music score was higher with CrystalisXDP than with the standard strategy. Nine patients (21%) categorized music above the random level (>5) on test 3 only based on mode with either of the strategies. In this group, CrystalisXDP improved the performances. For dissonance detection, 17 patients (40%) scored above random level with either of the strategies. In this group, CrystalisXDP did not improve the performances. Conclusions: CrystalisXDP, which enhances spectral cues, seemed to improve the categorization of happy versus sad music. Spectral cues could participate in musical emotions in cochlear implantees and improve the quality of musical perception.

## 1. Introduction

The effects of music on the brain extend far beyond hearing [1,2] and positively affect quality of life [3]. The stimulating properties of music not only promote the development of the auditory system in children [4,5] and increase the capacity of speech discrimination in noise [6], but also reinforce many cognitive capacities involved in communication skills and social integration [7,8]. For the hard of hearing, music is a valuable tool for training [7,8,9] and for exploring the hearing loss in a complementary manner to conventional audiometry [10]. It is a source of joy even in patients with profound hearing loss and a cochlear implant (CI), and this may explain their motivation to engage in musical rehabilitation programs [11].

In adult CI patients, music perception is severely deteriorated [12]. Recognizing melodies remains a difficult task and shows high interindividual variability (25% success versus 88% in normal hearing patients) [13]. This handicap is mainly attributed to the limitations inherent to CI sound coding and processing strategies [14,15,16]. In addition, auditory nerve survival in the implanted ears, which can be suboptimal, is directly related to the number of functional channels eliciting different auditory sensations, the electrical dynamic range, and also the capacity of benefiting from high rates of stimulations [17]. Alterations in central sound processing mechanisms caused by auditory deprivation may additionally contribute to this poor perception [18]. Despite these limitations, many implantees enjoy music [11,19], and some can also perform well, especially after training [20]. When evaluating CI patients for basic music characteristics such as rhythm, melody, and timbre, typically, poor pitch discrimination and melody recognition are described but a near-normal performance in rhythm perception is reported [21,22]. The existence of a few star patients and the effect of training on timbre perception and melody recognition suggest that some patients can extract spectral cues to compensate for a lack of pitch resolution, that central auditory processing is probably subject to plasticity in this field, and finally, that patients learn to enjoy music based on cues different from those used by normal hearing individuals [23]. This idea is supported by the observation that recognizing a melody is influenced by the timbre of the instrument in CI users [24]. These spectral cues depend largely on coding and the sound processing strategies [13,24]. Pitch resolution refers to the smallest pitch interval detectable by the patients, which is coded by the place (electrode position) and time (pulse rate and pattern) cues for each electrode and is related to the number of functional channels in the CI [17]. Spectral differences can generate different activation patterns across several electrodes, and their distinction requires complex peripheral and central mechanisms [25,26].

To improve sound quality delivered by CI, several interconnected issues should be tackled. Alteration in pitch perception severely deteriorates harmonies and musical lines [27]. This phenomenon is largely due to the modified cochlear tonotopy after CI [28] and the drastic reduction in functional channels (number of electrodes eliciting a distinctive pitch) entailing a significant loss of frequency resolution [29]. Attempts to increase the number of functional channels by current steering (simultaneous current delivery by adjacent electrodes with variable ponderation) have shown some improvement in speech performance [30] but cannot compensate for the reduced number of nerve endings in the cochlea.

Another issue in music listening with CI is the loss of spectral information. Better encoding the sound envelope and providing the temporal fine structure have shown their efficacy in enhancing bass frequency discrimination and higher musical sound quality [31]. However, these relatively new coding strategies encounter a pathophysiological barrier, which is the channel interaction and overload [18]. Indeed, delivering electrical pluses at a higher rate on a larger number of electrodes requires performant and numerous functional channels [32] that many patients do not have [33]. These channel interactions are largely responsible for inter-individual performance variability [33]. Improvement in the acoustic dynamic range is another paramount obstacle not only for understanding speech in noise, but also for enjoying music [34]. Indeed, delivering sound intensity nuances of daily life or music while disposing of a restricted range of tolerable sound intensities is problematic in many patients with a long history of hearing deprivation. With the increasing processing capacity of hearing aids, new sound processing algorithms such as nonlinear frequency compression and adaptative dynamic range optimization have been developed in the field of hearing aids [35,36,37], and some of these solutions have been more recently implemented in CI technology [34,38]. CrystalisXDP strategy (Oticon Medical, Vallauris, France) focuses on rendering the spectral details of the entering signal with a lower distortion than the standard “main peak interleaved sampling” (MPIS) strategy. In addition, it provides possibly more comfortable listening through an adjustable compression system [38]. In a previous study, this sound processing strategy seemed to enhance speech perception in quiet and noise [38]. Its effect on music perception has not been evaluated to our knowledge.

The present study focuses on the emotional response to music, which is the most important aspect of everyday life music experience. Tempo and mode were found to be the most robust factors inducing joy and sadness in listeners [39]. A given musical piece will be perceived to be happier when played faster, and in major rather than in minor mode. Although the perception of tempo raises no difficulty in CI, the perception of mode remains a challenging issue. In contrast to the major mode, minor music contains intervals such as minor third intervals, which induce significant roughness or dissonance in the auditory filter [40]. Accordingly, we hypothesized that, with a poor pitch resolution, CI patients would have difficulty distinguishing happy from sad music using spectral cues, but that reducing the spectral distortion would enhance this capacity. The goal of this study was to evaluate the effect of reducing spectral distortion with the CrystalisXDP sound processing program on the ability of CI patients to distinguish happy from sad music based on rhythmical and/or modal cues, and to confront this performance to their subjective musical experience.

## 2. Materials and Methods

Twenty-one patients were included in this prospective double-blind and crossover study. Inclusion criteria in this study were the following: adult patients with bilateral profound hearing loss, unilateral or bilateral cochlear implants with at least one year of experience, Digisonic CI and Saphyr 2 sound processor (Oticon Medical) in their monaural or binaural versions, and a dissyllabic word discrimination score (WDS) >20% with CI alone.

Among the 48 patients corresponding to these criteria in our center, we excluded 21 (44%) who did not wish to participate, 6 (13%) who had moved from our region and were lost to follow-up, and 1 who had poor speech recognition (WDS with CI alone: 14%). Twenty-one patients were included: five had a unilateral Digisonic DX10^®^ (bearing 15 electrodes); t wore a unilateral and one a bilateral Digisonic SP^®^ (20 electrodes); and three were rehabilitated by a binaural Digisonic^®^ CI (12 electrodes on each side).

The protocol was reviewed and approved by the institutional ethical committee (CCP grand Est III). All patients were clearly informed and provided their oral and written consent for this study.

### 2.1. Study Design

At inclusion, the patient was examined, underwent a hearing test, and filled in a questionnaire on past and current musical experiences. The visit ended with a standard fitting session by the audiologist. Two programs (P1 and P2) were downloaded into the processor. During the study, the fitting parameters (frequency allocations, loudness) remained the same for the 2 strategies. CrystalisXDP and standard MPIS strategy were randomly assigned to P1 and P2 program slots in a double-blind manner to the patient and to the investigator who tested the hearing performances and the musical experience. The patient was asked to use P1 for one week. A second visit was then programmed. The patient participated in a musical test and responded to a questionnaire pertaining to experience with P1. Subsequently, P2 was activated. One week later, P2 was evaluated with the same tests. In bilateral and binaural cases, the programs were applied to both ears.

### 2.2. Population Characteristics

Twelve women and nine men participated in the test (Table 1). The mean age of the group was 55 ± 2.7 years (23–74). All presented with postlingual deafness. Patients had been implanted for a mean duration of 8 ± 1.2 years [3,4,5,6,7,8,9,10,11,12,13,14,15,16,17,18,19] before inclusion. The hearing deprivation period before implantation was 9 ± 3.1 years [1,2,3,4,5,6,7,8,9,10,11,12,13,14,15,16,17,18,19,20,21,22,23,24,25,26,27,28,29,30,31,32,33,34,35,36,37,38,39,40,41,42,43,44,45,46,47,48] and the mean age at implantation was 47 ± 2.6 years [19,20,21,22,23,24,25,26,27,28,29,30,31,32,33,34,35,36,37,38,39,40,41,42,43,44,45,46,47,48,49,50,51,52,53,54,55,56,57,58,59,60,61,62,63,64,65].

Seventeen patients (81%) had a unilateral CI (nine right and eight left), three (14%) had a binaural CI, and one (5%) had a bilateral CI. All patients wore their CI more than 12 h per day. Seven patients (33%) with a unilateral CI had a contralateral hearing aid. Before inclusion, 15 patients used CrystalisxDP and 6 used the standard MPIS strategy.

Etiologies of hearing loss were idiopathic in 11 patients (52%), Meniere’s disease in 1 (5%), congenital in 4 (19%), advanced otosclerosis in 2 (10%), and traumatic in 2 cases (10%).

The ipsilateral pure-tone average (PTA) was 108 ± 8.8 dB before implantation and 39 dB ± 3.1 in free-field with CI. The aided contralateral PTA was estimated as 79 ± 11.2 dB (*n* = 21) with no response above 1 kHz. The WDS was 6.5 ± 9.88% without CI and with lipreading only, 58.6 ± 22.01 with CI only, and 78.3 ± 19.25 with CI + lipreading.

### 2.3. Coding and Sound Processing Strategies

The main peak interleaved sampling (MPIS) strategy was used as the standard strategy in this study [41]. The speech processor (DigiSP) uses a Fourier Frequency Transform (FFT) to extract frequency peaks from the input signal spectrum in the 195–8003 Hz range. Available intracochlear electrodes, or channels (ranging from to 9–20 in this study), are selected for assignment of frequency bands to cover the 195–8003 Hz range using monopolar constant current stimulation. The signal level in each of the bandpass filters is assigned to the active electrodes. Loudness is coded by pulse duration, and pulse amplitude remains constant over time. Active electrodes associated with the highest signal level (spectral maxima) are stimulated in a basal to apical order. The number of transmitted peaks can be modified (default setting: 10 transmitted peaks out of 20 extracted peaks). The number of channels to be stimulated at each cycle is predetermined during fitting. Electrical stimulation rates range from 150 to 1000 pulses per second per electrode (pps/e). The default factory setting is 600 pps/e. Patients in this study used default settings. Only the number of available electrodes changed from one patient to another.

**Figure 1:**
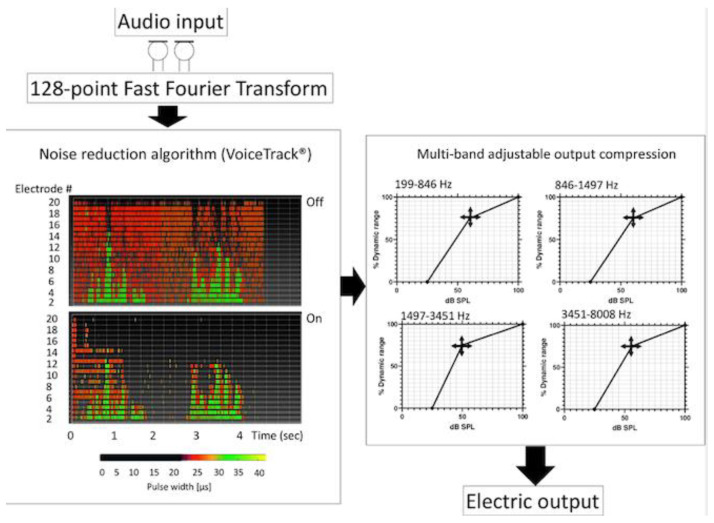
**Functional Structure of CrystalisXDP.** The system extracts the spectral features of the acoustic input by a 128-point Fast Fourier Transform (FFT). A noise reduction algorithm (VoiceTrack) based on spectral subtraction is then applied to enhance the spectral contrast. The 2 diagrams in the VoiceTrack panel show the simulated electrodograms of a human speech sample (dissyllabic word, 4 s), before (top) and after processing (below), generated by an in-house Oticon Medical simulation program as an example. Finally, the multi-band output compression provides adjustable output levels (*Y*-axis in % of electric dynamic range) as a function of acoustic input (*X*-axis, dB SPL) in 4 frequency bands.

**Figure 2:**
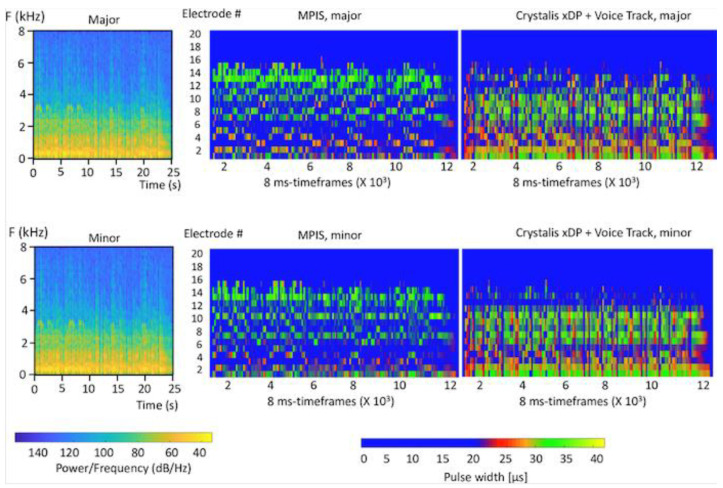
**Spectrograms of the acoustic input, and electrodograms with standard MPIS and Crystalis xDP for 2 samples from test 3 with the same melody in major and minor modes**. Spectrograms and electrodograms were simulated on Mathlab software using the same algorithms used in the processors by an in-house Oticon Medical program. For electrodograms, vertical axis shows electrode numbers (from 20 at the apex to 1 at the base) and the horizontal axis shows the number of analysis frames for the total duration of the sample (25 s). Each pixel represents an 8 ms frame sliding every 2 ms. Color codes represent pulse width (µs) coding for intensity for electrodograms and power/frequency (dB/Hz) for the spectrograms. Both strategies produced different electrodograms for minor and major modes. Crystalis xDP showed a richer electrodogram with more spectral cues. Differences between minor and major modes were translated by both temporal and spectral differences (i.e., different activation patterns across channels and within channels).

The digital signal processing of CrystalisXDP (Figure 1 and Figure 2) is an evolution of the standard MPIS strategy specifically designed to enhance speech discrimination. It incorporates a multichannel back-end output compression function designated as XDP [38]. The Crystalis coding strategy enhances the FFT analysis by a window analysis in order to suppress artifacts and to extract not only the most salient but also the most relevant peaks to speech discrimination. The signal input spectrum is then processed by a noise reduction algorithm (Voicetrack^®^) that is based on a human voice reconnaissance and spectral subtraction. The signal is sent to the XDP transfer function module, which provides an adjustable compression of the electrical dynamic range as a function of the acoustic dynamic range. The knee point can be adjusted independently for four frequency bands: 195–846; 846–1497; 1497–3451; and 3451–8000 Hz, which groups electrodes with a similar energy spectrum for speech. Ninety-five percent of the speech information falls in the area under the knee point in each ambience considered. In this population, a medium preset for the knee point was used (average sound intensity at 70 dB SPL). In comparison to the standard MPIS strategy, CrystalisXDP improves the selection of the most relevant spectral peaks; it enhances the spectral contrast of the signal by a noise-reduction algorithm after the FFT analysis; and finally, it provides fine adjustment of the input–output compression function in order to contain everyday life sounds in a comfortable range.

### 2.4. Clinical Data

Clinical data regarding hearing loss (etiology, duration of deprivation, age at implantation) and audiometry data (pure-tone and speech performances with speech reception threshold, SRT and word discrimination score, WDS) before and after implantation were recorded.

Audiometry was performed with a calibrated audiometer (AC40^®^, Interacoustics Inc., Middelfart, Denmark) in a standard audiometric booth. Preoperative tests were conducted with a headset. Postoperative tests were conducted in free-field conditions with 2 frontal loudspeakers and contralateral masking (headset and white noise). SRT and WDS were evaluated by French Fournier dissyllabic lists. WDS was tested at 60 dBA (SPL).

### 2.5. Questionnaires

The musical questionnaire was a simplified version of Munich Music Questionnaire (MMQ, 42) to limit the duration of each session. The questions concerned the musical experience in daily life through the average time of daily music listening, sound quality, instrument recognition, importance or implication of musical activities in the past and present (Table 2), and the sound and music perception by their CI before inclusion (Table 3). For this question, CI experience was compared to the period before implantation with still some degree of functional hearing for the progressive congenital or acquired diseases.

### 2.6. Music Test

We designed a music trial composed of 4 tests with increasing difficulty. The first 3 tests assessed emotional perception through music. In each of these tests, 6 melodies were played on piano in major (happy) and in minor (sad) modes, representing a total of 12 musical samples of 25 s each. The melodies were unknown to the general public in order to avoid cultural references. The melody line was accompanied by 1–4 note chords. Stimuli were equally tempered MIDI piano notes. All samples were recorded with a 44.1 kHz sampling rate at 16-bit depth. The participant could listen to these samples in a free order and as many times as desired. The subject was asked to categorize these samples as happy or sad with a forced two-choice task. No feedback was given. In the first test (easiest), in addition to the mode difference, happy samples were played faster than sad excerpts with a large difference in tempo (vivace, 140 beats/min for happy versus andante, 80 beats/min for sad). In the second test (intermediate), there was only a small difference in tempo (moderato, 100 versus 90 beats/min). In the third test (most difficult), the tempo was identical (moderato, 90 beats/min) and only the mode difference could allow the distinction. Test 4 evaluated the dissonance perception. Ten melody samples, with (*n* = 5) or without (*n* = 5) dissonance, were presented and the patient had to categorize them as “dissonant” or “harmonious”. Melodies had the same characteristics as in the first 3 tests and were played with a moderato tempo (100 beats/min.). All tests were finally marked out of 10. The test interface was a laptop computer screen (Powerpoint 2010, Microsoft Inc. Redmond, VI, USA) where the patient could click on the musical sample to listen and to drag-and-drop the file into the proposed categories represented by happy and sad emojis. Samples were presented on 2 frontal loudspeakers (Sony, SRS-Z510, Tokyo, Japan) at a comfortable level judged by the patient. All tests were conducted in CI-only mode. In patients with residual hearing, the hearing aid was deactivated, and a sound reduction ear plug was placed in the ear. The patient used the interface independently and was only assisted by the investigator for technical issues.

At the end, an auto questionnaire allowed the participant to rate the clarity of sound, the enjoyment of the melody, and the ease of each test (Likert scale 1–10), and to answer to the question, “which program did you prefer?” in a blinded manner (program 1 or 2, at the end of the second session).

### 2.7. Statistical Tests

Data were managed with Excel software (Office 2010, Microsoft Inc. Redmond, VI, USA) and Graphpad prism (v.6, Graphpad Inc., San Diego, CA, USA). Continuous variables were presented as mean ± standard error of mean (SEM) [min.-max.] and nominal variables were noted as *n* (%). Comparison of continuous parameters in 2 groups was studied by paired or unpaired t-tests. Continuous variables in multiple groups were tested by one- or two-way ANOVA. Music test scores were compared to the random level (score 5 out of 10) for each test by a one-sample t-test. A *p*-value < 0.05 was considered as significant. Linear regression analysis was conducted by F-test for the slope of the regression line and R for goodness of fit. Correlations were considered significant when R > 0.5 and *p* < 0.05. Test–retest reliability was tested by Cronbach’s alpha. A value in the range of [0.8–0.9] was considered as good and >0.9 as excellent. To control for the effect of the usual strategy used by the patients in their music test performances, a mixed-model analysis was used to compare the results of the 4 music tests with the CystalisXDP versus MPIS program as a function of their usual strategy. A separate model was mixed employed for the global music score.

The population size was estimated for test–retest reliability by setting α = 0.05, β = 0.1, k (number of test items) = 4, the value of Cronbach’s alpha at null hypothesis = 0, and the expected value of Cronbach’s alpha = 0.75. The required number was evaluated as 17 subjects according to Bonnett [42] and increased to 21 to account for potential loss to follow-up at the retest.

## 3. Results

### 3.1. CI and Sound Processing Strategies

The number of active electrodes was 15 ± 0.7 (*n* = 21): 11 ± 2.5 for patients with Digisonic DX10 (*n* = 5), 18 ± 1.5 for unilateral Digisonic SP (*n* = 12), 12 on each side for binaural CI (*n* = 3), and 16 and 18 for the bilateral Digisonic SP. Before inclusion, 6 patients were fitted with the standard program (MPIS) and 15 already used CrystalisXDP. Patients using CrystalisXDP before inclusion performed similarly to those with a standard program as assessed by WDS (78 ± 6.5% *n* = 15, versus 60 ± 13.9, *n* = 6, not significant, unpaired t-test followed by Bonferroni). Speech performances were not related to the number of active electrodes in this group (WDS: 83 ± 5.8%, *n* = 15, versus 48 ± 10.5, *n* = 6, respectively, not significant, unpaired t-test, followed by Bonferroni correction).

### 3.2. Musical Experience

At inclusion, the questionnaire revealed that music was important in the daily life of this group (average Likert score 3.6 ± 1.20, with 18 patients (86%) scoring >3 out of 5, Table 2). The implantation did not change the frequency of music listening (response to “How often?”, not significant, chi-2 test), or the type of music (not significant, chi-2 test). The majority (18, 86%) continued to listen for pleasure (Table 3) and practiced active music listening (17, 81%). While most declared being capable of recognizing a known melody (18, 86%), the musical style (15, 71%), and even the lyrics (15, 71%), only a few declared being capable of detecting a wrong note (6, 29%), singing in tune (5, 24%) or singing in public (2, 10%) underlining the inherent CI limitations in frequency discrimination.

CI negatively impacted music activities in this group. After implantation, many patients stopped musical activity such as music lessons (6 out of 7), playing an instrument (3 out of 6) or singing (3 out of 9). However, most declared training themselves with music after CI (13, 62%).

### 3.3. Music Test

**Figure 3:**
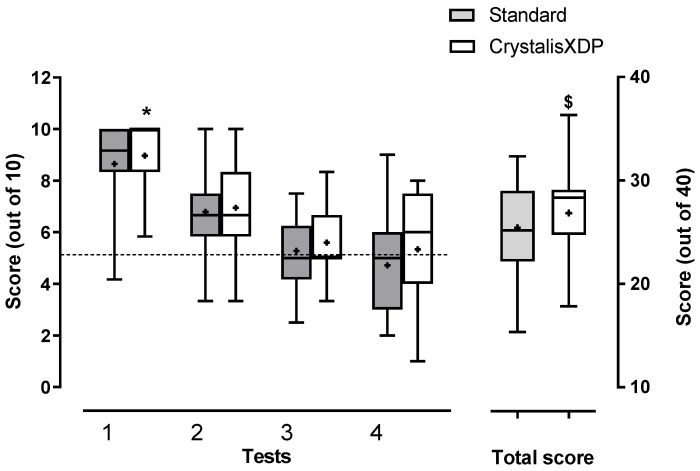
**Scores for music tests with standard (MPIS) and CrystalisXDP sound processing strategies.** Each test was marked out of 10, and the total score out of 40. Bars represent mean ± SEM (*n* = 21). Scores decreased with the difficulty level (*: *p* < 0.001, mixed model analysis). Patients performed better with CrystalisXDP than with standard program (*p* < 0.05) regardless of their usual strategy (effect not significant). Total scores were also higher with CrystalisXDP than with MPIS regardless of the patients’ usual strategy ($: *p* < 0.05, mixed-effects analysis). Box and Whiskers plot represents first and third quartiles, median, and range. Mean is depicted by (+). Dashed line represents chance level.

Scores decreased with increasing levels of difficulty from tests 1 to 4 for both CrystalisXDP and standard programs (Figure 3). Scores for tests 1, 2 and 3 were above chance level (8.81 ± 0.25 for test 1, *p* < 10^−4^, 6.87 ± 0.25, for test 2, *p* < 10^−4^, and 5.43 ± 0.20, *p* < 0.05 for test 3, *n* = 42, one-sample test). In contrast, the average score for test 4 was not different from the chance level (5.02 ± 0.31, *n* = 42, not significant, one sample test, Figure 3).

The short period of adaptation could have advantaged CrystalisXDP over the standard program in those who already used CrystalisXDP and represented the majority (15 out of 21). A mixed-model analysis (restricted maximum likelihood approach) comparing the results for music tests 1 to 4 with CrystalisXDP and standard strategies in patients who regularly used CrystalisXDP versus those who regularly benefited from the standard program showed a significant effect of the test levels (DFn = 3, DFd = 76, F = 32.15, *p* < 0.001) and the strategy during the test (higher scores for CrystalisXDP versus standard, DFn = 1, DFd = 76, F = 5.76, *p* < 0.05). However, the usual strategy used by the patients before inclusion did not have a significant effect on the test results (CrystalisXDP versus standard, DFn = 1, DFd = 76, F = 0.12, not significant). There was no interaction between these factors (test level*tested strategy: DFn = 3, DFd = 76, F = 1.52, *p* = 0.214; test level *initial strategy: DFn = 3, DFd = 76, F = 1.31, not significant; Tested strategy*usual strategy: DFn = 1, DFd = 76, F = 0.021; not significant; test level*tested strategy*initial strategy: DFn = 3, DFd = 76, F = 0.081, not significant). A Tukey’s multiple comparison test applied to this model showed a higher level of scores for test 1 in comparison to all other tests (*p* < 10^−4^), a higher score for T2 in comparison to test 4 (*p* < 0.001), and higher scores for T3 versus T4 (*p* < 0.05).

As assessed by the total score, patients also performed better with CrystalisXDP than with the standard program regardless of their usual strategy (mixed-effects analysis, DFn = 1, DFd = 38, F = 4.98, and *p* < 0.05 for the effect of the tested strategy; F = 0.644, not significant for the effect of usual strategy, and F = 0.046 not significant for tested strategy*usual strategy, Figure 3). Higher scores with CrystalisXDP suggested that patients exploit some spectral-based cues in addition to the rhythm to distinguish between happy and sad music.

There was no statistical difference between the total scores at the first and second sessions, suggesting that there was no effect of order (global scores 30.5 ± 5.19 vs. 31.2 ± 5.23, respectively, mean of differences: 1.52, not significant, paired-*t*-test, *n* = 21). The test–retest reliability of the total score was good between the two sessions (Cronbach alpha = 0.87, average R = 0.77).

Musical background was significant in this population. Ten patients used to sing in their childhood (47%). Among these, five continued singing during adulthood and even after CI. Seven declared playing an instrument in their childhood: drums (*n* = 1), flute (*n* = 1), piano (*n* = 3), accordion (*n* = 1), and clarinet (*n* = 1). Only four pursued their hobby as an adult. Five singers also played an instrument. Singing before CI tended to improve scores regardless of strategy (*p* = 0.05, 2-way ANOVA, Table 4), but there was no effect of playing an instrument or training with CI on the scores (not significant, 2-way ANOVA).

**Figure 4:**
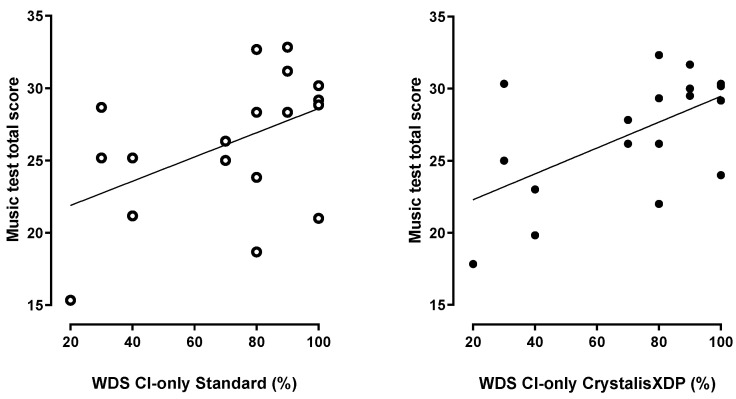
**Correlation between musical test total scores and word discrimination scores (WDS) with cochlear implant (CI) only with standard (MPIS) and CrystalisXDP sound processing strategies.** WDS tended to be correlated with total scores in standard condition (right panel, Y = 0.08 * X + 20.2, R = 0.47, *p* < 0.05, F test) and was significantly correlated to total scores in CrystalisXDP condition (left panel, Y = 0.09 * X + 20.5, R = 0.58, *p* < 0.01, F-test).

**Figure 5:**
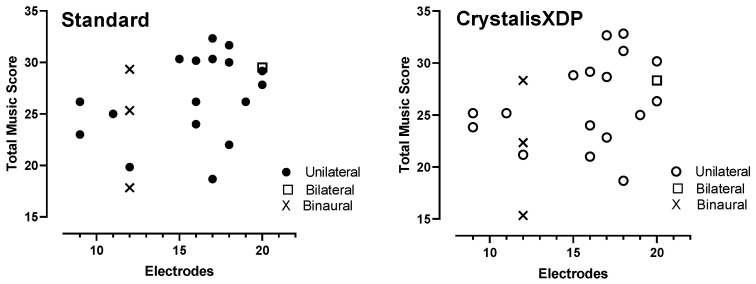
**Total music scores as a function of the number of active electrodes with standard (MPIS) and CrystalisXDP strategies**. Bilateral and binaural cases are depicted with the number of electrodes in one ear (20 and 12, respectively).

Total music scores were correlated with WDS (Figure 4). Total music scores appeared to be influenced by the number of active electrodes. Although there was no correlation between the number of electrodes and the total score (Figure 5), patients with more than 15 electrodes (*n* = 14) performed better with CrystalisXDP sound processing programs (28 ± 5.89, *n* = 7 for patients with <15 electrodes versus 33 ± 3.93, *n* = 14, t(19) = 2.18, *p* = 0.042, unpaired t-test). With the standard MPIS program, this difference also tended to be significant (26.6 ± 4.12, *n* = 7 versus 30.8 ± 5.10, *n* = 14, t(19) = 2.07, *p* = 0.052, unpaired *t*-test).

Total scores obtained by patients with unilateral CI were not different from those with binaural or bilateral CI (31.8 ± 4.57, *n* = 17 versus 29.6 ± 5.04, *n* = 4, with CrystalisXDP, and 29.5 ± 7.68 versus 28.5 ± 6.14 without CrystalisXDP, not significant, unpaired *t*-test). Patients with bimodal hearing did not perform better than those with one or 2 CIs in this population (29.1 ± 3.81, *n* = 7 versus 25.5 ± 1.55, *n* = 14, respectively, with standard program, not significant, unpaired *t*-test, data not shown for CrystalisXDP). Similarly, patient who reported musical training during rehabilitation with CI did not perform better than others according to the total score or the scores obtained for each test (data not shown). Patients performed well at tests 1 and 2 and these scores were highly correlated, suggesting the prominence of rhythmical cues even for small differences in tempo in test 2 (Y = 1.00 + 0.67 X, R = 0.73, *p* < 0.001, and Y = −0.31 + 0.81 X, R = 0.67, *p* < 0.001 for standard and CrystalisXDP were Y: test 2 and X: test 1).

In contrast, only nine (43%) patients could categorize above the random level (score > 5) in test 3 (sad versus happy based only on mode) with the standard or CrystalisXDP programs (average scores 6.4 ± 0.59 and 6.9 ± 0.93, respectively, *p* < 0.001, one-sample test for both). In this group, CrystalisXDP, significantly improved the score in comparison to the standard strategy (*p* < 0.05, paired *t*-test, followed by Bonferroni correction). Similarly, only a few patients could distinguish dissonance above chance level (score >5 at test 4): 6 (29%) with standard program (average score 7.0 ± 1.27, *p* < 0.05, one-sample test) and 11 (52%) with CrystalisXDP (average score: 7.0 ± 1.00, *p* < 0.0001, one-sample test). In this group, CrystalisXDP did not improve the scores (not significant, paired *t*-test, followed by Bonferroni correction).

Performances for tests 3 (sad/happy only based on mode) and 4 (dissonance) were similar (5.4 ± 0.24 versus 5.0 ± 0.40, respectively, *n* = 21, average of two programs, unpaired *t*-test, not significant), but not correlated (data not shown), suggesting that these two tasks explored different domains. The duration of the hearing deprivation influenced the scores for test 3: patients with a score >5 with CrystalisXDP had a hearing deprivation period <10 years in all cases (*n* = 8), while those who performed poorer had longer deprivation periods (6 out of 12 with deprivation >10 years, *p* < 0.05, chi-2 test). Performances in test 4 were not related to hearing deprivation period (data not shown). Additionally, scores >5 in tests 3 and 4 were not related to age, sex, number of active electrodes, contralateral hearing aid, or previous training (data not shown).

These poor performances contrasted with the questionnaire results in which the majority (18, 86%) declared hearing the melody most (or best) (Table 3). The performances in tests 3 and 4 were not higher in those who declared detecting wrong notes than others (data not shown).

**Figure 6:**
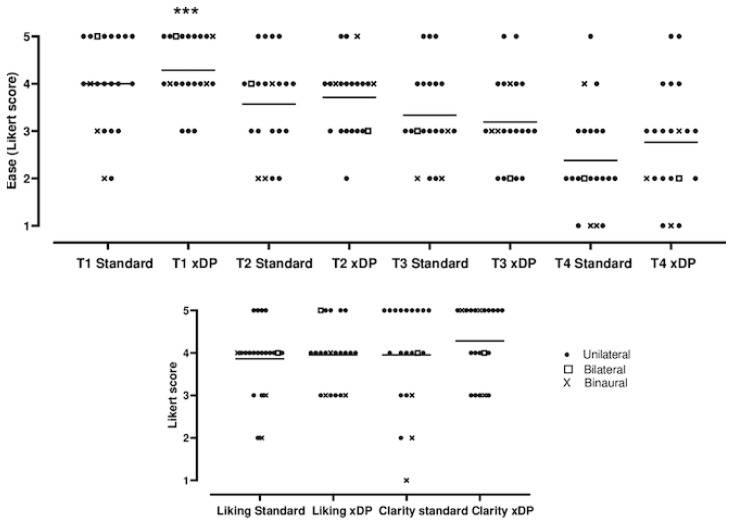
**Musical test ratings in terms of ease, clarity and melody liking.** Patients scored each item on an auto questionnaire at the end of each test on a Likert scale (1 to 5). Symbols (***) represent individual values (*n* = 21) and bars represent mean. Ease scores decreased with the difficulty level, but programs (standard or MPIS versus CrystalisXDP) did not influence ratings (*p* < 0.001 for test levels and not significant for programs, 2-way ANOVA), unpaired *t*-test versus standard.

The subjective ease scores decreased with the level of difficulty (Figure 6). Sound processing programs did not influence the ratings of ease, sound clarity or liking (Figure 6). There was a significant correlation between the total music score and the level of ease rated by the participant for the first test (first trial: Y = 2.58 + 0.17X, R = 0.5, *p* < 0.05, second trial: Y = 0.45 + 0.33X, R = 0.6, *p* < 0.01, F-test, X: score, Y: level of ease), but for more difficult levels involving modes and dissonances (tests 2 to 4), this correlation did not exist (data not shown). Clarity and liking ratings were not correlated with total music scores (data not shown) and were not modified by the program (not significant, unpaired *t*-test, Figure 6).

Interestingly, test 3 (happy versus sad based on mode) was rated as easier than test 4 regardless of the program (3.3 ± 0.16 for test 3 versus 2.6 ± 0.20 for test 4, average scores for 2 programs, *n* = 21, *p* < 0.01, paired t-test), while the performances were similarly poor for both tests. Finally, most patients (*n* = 16, 76%, *p* < 0.05, binomial test) preferred CrystalisXDP to the standard MPIS. Among patients (*n* = 15) who used CystalisXDP before the study, 12 kept their usual program and 3 chose the standard program. In the group using MPIS regularly (*n* = 6), three conserved their program and three switched to CystalisXDP.

## 4. Discussion

In this study, we showed that music represents a significant daily activity for cochlear implantees. Our original music test, which assessed the hearing performances and explored the emotional aspect of the music, yielded a total score correlated to word discrimination score. It had a good test–retest reliability and did not have a floor or ceiling effect. It was positively influenced by a higher number of active electrodes. As expected, the test revealed a good detection of rhythmical cues but poor performances in detecting dissonances and musical modes. CrystalisXDP improved the musical test results based on both rhythm and spectral cues. Since MPIS and CrystalisXDP have the same basic coding strategy providing the same rhythmical information, and the fitting parameters were identical for both strategies, the results suggest that this improvement is related to modifications in spectral cues.

Musical experience is difficult to describe and analyze since it deals with several intricate factors such as rhythm, pitch, timbre, melody, cultural references, and complex capacities, such as musical sophistication [44]. The latter parameter is defined by the frequency of exerting musical skills or behaviors and the ease, the accuracy or the effect of musical behaviors, and a varied repertoire of musical behavior patterns can be a source of inter individual variability in music tests [44].

Most of the reported music tests evaluate basic features such as pitch, timbre, and rhythm perception [19,45,46]. However, considering the gap between poor musical hearing performances with a CI and a relatively high music enjoyment [47,48,49], it is interesting to explore higher levels of music perception such as emotions since it can a lead to better understanding of coping mechanisms and neural plasticity in cochlear implantees [50,51].

The effect of Western musical modes on emotions is well known and appears to be effective even in individuals with little or no musical background [for review, see 52]: the major mode evokes dynamism, joy, hope, force and tenderness, and oppositely, the minor mode elicits passivity, despair, sadness, pain, mystery and obscurity. To control the overall difficulty of the trial, we organized the tests in a gradually increasing order of complexity. The rhythmic cue, known to be largely exploited by the CI patients [53], was employed to mitigate the difficulty of the pitch and mode discrimination. As expected, the performances and the level of ease rated by the participants decreased with a lower contribution of rhythm in the categorization. Without this hint, the average score dropped from excellent to chance level for tests 3 (happy versus sad only based on mode) and 4 (dissonance in a melody). This poor performance was in line with the questionnaire in which only 29% of the patients declared being capable of detecting a wrong note. It is noteworthy that CrystalisXDP, which improves spectral cues but provides rhythmical information similar to MPIS, enhanced the happy versus sad categorization performances based on both musical modes and rhythmical information. Previous reports have shown that in cochlear implantees, both place (i.e., electrode position in the cochlea and its assigned frequency band) and temporal cues (i.e., stimulation pulse pattern and rate) are closely related to each other for pitch perception [54,55]. In our study, while place cues remained the same, temporal cues were modified through spectral modifications by CrystalisXDP. The optimization of the temporal cues might influence the pitch perception and provide a possible explanation for the enhancement of sad versus happy categorization.

However, interestingly, a few patients performed relatively well (scores > 5) for these tasks despite the inherent limitations of CI. Better scores for test 3 (happy versus sad based on only mode) were obtained by patients who had a short time of hearing deprivation (<10 years), suggesting the need for an efficient auditory central pathway in music processing [16]. Scores for tests 3 and 4 were not correlated, while scores for tests 1 and 2 (categorization mainly or partly based on rhythm) were highly correlated. This observation suggests that musical modes may involve a different auditory processing task than the detection of a dissonance in a melody. Another important factor, which may explain high performances in tests 3 and 4, is the above-average spectral and pitch resolution related to a higher neural survival in the implanted ear. The quantity of preserved neurons directly influences the number of functional channels, the channel interactions, and the neural capacity to be stimulated at high rates [17,31,32,33,56].

The distinction of consonant from dissonant notes from a musical instrument or human voices is directly related to the interval between their fundamental frequencies and mainly detected at the cochlear level [57,58]. A dissonant note with fundamental frequency (F0) too close to the reference note to be resolved by the cochlea produces a rapid variation in total amplitude and a sensation of roughness or beating which can be evidenced on the spectrogram [59]. A dissonant note easily distinguishable by the cochlea from the reference has component frequencies that cannot aggregate with those of the reference note producing an inharmonic spectrum. The participation of central auditory processing in this distinction has been suggested based on observation of subjects with amusia [59], but the exact role of peripheral auditory system and the auditory centers are extremely hard to separate in this process. To this end, CI patients represent an interesting pathophysiological model. Observations on CI patients with contralateral normal hearing are in line with this mechanistic explanation. CI patients appear to be sensitive to dissonance by the perception of roughness, and the information related to the temporal envelope plays an important role in distinguishing harmonicity from dissonance [40]. In our study, reducing the spectral distortions without altering the rhythmic information by CrystalisXDP sound processing strategy improved total scores, leading to the hypothesis that by providing discrete cues on roughness and beating, it could enhance global music perception. This phenomenon may be explained by the reduction in spectral smearing and undesired channel interactions in CI patients. Spectral information directly influences the temporal coding within channels. This possible explanation is in line with the observation that reducing the number of harmonics increases the musical enjoyment in both normal-hearing and CI subjects [60].

Despite their poor performances in tests 3 and 4, patients attributed an above-average score to the clarity and the liking of the melodies, and this discrepancy underlines the difference between performance and enjoyment, an observation that has also been reported by others [45,46]. With time, CI patients develop other musical esthetic criteria, and choose types of music which are easier to listen to (more rhythmical cues, less polyphony, and harmonics) as coping strategies [61]. To enjoy music with CI, postlingually deaf patients need time and effort to gather musical experience with new sensations and auditory landmarks. Pleasant music is a skilled mix of predictable events, which drive expectations, and sparse unpredictable developments leading to surprises, and these expectations are related to the experience of musical pleasure [62,63]. Alterations in timber perception and low pitch resolution deteriorate the melody reconnaissance in CI patients [13] and probably also the predictability. With training, these auditory expectations and surprises can be developed in CI patients [7,8,9]. Another issue is that musical pleasure seems to increase with stimulus complexity (e.g., musical lines, harmonics, timber) up to an intermediate level, and then to decrease with even more complex sounds [64]. Achieving such a level of performance to detect complexity appears possible in some CI patients, since in our population, 9 declared listening to classical music and 5 to opera, reputed as relatively complex, and 15 declared being capable of even comparing performances. However, this ability probably requires a high number of functional channels in the cochlea and a performant central auditory pathway [24,65].

Many variables, such as number of active electrodes, insertion depth, or duration of hearing deprivation may have an impact on the music perception in CI patients [66] and explain the heterogeneity of the results. However, when attempting to control all variables in a very homogeneous population, one might argue that the observations do not apply to other groups of CI patients and the effect is marginal. In addition, one might oppose the fact that other variables such as sex, age, body laterality, ethnicity and cultural background could still interfere. Moreover, it would be difficult, if not impossible, to control parameters such as electrode insertion depth and electrode position or even musical background and experience in such a population. Consequently, we compared sound processing strategies in a paired cross-over design to limit the potential effect of these factors in the outcome. Despite the heterogeneity, which corresponds to the every-day audiology practice, we could observe a quite significant effect of spectral cue enhancement on the music scores. Using only one or both ears could influence the results. However, interestingly, total scores obtained by patients with unilateral CI did not differ from those with binaural or bilateral CIs. Patients with bimodal hearing had marginal acoustic hearing and were tested in CI-only mode; they did not perform better than those with one or 2 CIs in this population. This is consistent with the experimental conditions, which did not disadvantage monaural patients (twin frontal loudspeakers).

In our study, the adaptation period to new sound processing strategies was relatively short. This could have masked the effect or created a bias. However, CrystalisXDP is not a radical change in strategy in comparison to the standard program. It improves the already installed strategy by a better selection of spectral peaks to code, by increasing the spectral contrast, and by fine-tuning the output compression. There is no change in the frequency-place function, frequency band allocation, the loudness or even the basic strategy, which is the MPIS. A previous publication on this sound processing algorithm had shown a rapid adaptation of the patients with significant improvements of WDS in 30 days [38]. This is consistent with the improvement of music scores with CrystalisXDP, which were correlated with WDS in this study. The short adaptation period could have advantaged CrystalisXDP in the majority who used this strategy before inclusion. However, a mixed-model analysis showed that the strategy used regularly before the inclusion did not affect the results.

To our knowledge, there is no validated test for evaluating the emotional aspects of music or musical experience in cochlear implantees. The Munich Music questionnaire has not been validated but was previously published as a relevant tool to evaluate musical perception in CI patients [42]. This questionnaire appeared to provide coherent and consistent results in cochlear implantees from different countries and cultural backgrounds [42,67,68,69]. This lack of validation imposes precaution in the interpretation of the results related to this tool. In contrast, Likert scales have been largely used as a validated method for the psychometric evaluation of music perception [70] and auditory handicap [71] and provided coherent information regarding the ease of the tests.

In conclusion, the categorization of happy versus sad music samples only based on musical mode or the distinction of melodies with dissonant notes from harmonious ones did not exceed the chance level. CrystalisXDP, which enhances spectral cues, improved performances in the categorization tasks where some rhythmic information was added to the musical mode. This observation, together with the music experience through questionnaires, suggests that CI patients exploit not only rhythmical indications, but also spectral cues to enjoy music and that tests based on intervals, rhythm and melody recognition cannot fully comprehend these cues. Further work on these potential spectral cues will guide the development of next generation sound processing strategies.

## Figures and Tables

**Table 1:** **Subject demographics.** Age, hearing deprivation and CI experience are expressed in years. Hearing deprivation began by the abandonment of the ipsilateral hearing aid. Process.: Type of Saphyr processor, CI Exp: Cochlear implant experience, F: female, M: Male, L: Left, R: Right, BIN: binaural, BIL: bilateral. Number of active electrodes/total electrodes in BIN and BIL cases are indicated as Right + Left.

ID#	Sex	Age	Etiology	Hearing Deprivation	CI Exp.	CI Side	Process.	Active/Total Electrodes	Initial Strategy
1	M	50	Idiopathic	3	5	L	SP	18/20	Crystalis
2	M	53	Trauma	1	7	R	SP	18/20	Crystalis
3	M	47	Congenital	1	3	L	SP	18/20	Crystalis
4	M	23	Congenital	1	4	R	SP	20/20	Crystalis
5	F	51	Idiopathic	1	4	L	SP	17/20	Crystalis
6	F	62	Idiopathic	1	19	L	SP	9/15	Crystalis
7	M	67	Idiopathic	1	11	L	CX	15/15	Crystalis
8	M	62	Idiopathic	48	3	L	SP	19/20	Crystalis
9	F	67	Idiopathic	37	17	L	CX	12/15	MPIS
10	F	58	Otosclerosis	14	19	R	CX	9/15	MIPS
11	F	74	Otosclerosis	1	10	L	SP	16/20	MPIS
12	M	63	Idiopathic	1	4	R	SP	20/20	Crystalis
13	F	54	Idiopathic	1	5	BIN	SP	12/12 + 12/12	Crystalis
14	F	55	Idiopathic	8	6	BIN	SP	12/12 + 12/12	Crystalis
15	M	69	Meniere’s	29	6	L	SP	16/20	Crystalis
16	F	56	Congenital	28	16	R	CX	11/15	MPIS
17	F	47	Idiopathic	3	3	R	SP	17/20	Crystalis
18	F	44	Idiopathic	2	2	L	SP	16/20	Crystalis
19	F	38	Congenital	1	6	BIN	SP	12/12 + 12/12	MPIS
20	F	38	Idiopathic	3	4	BIL	SP	16/20 + 18/20	MPIS
21	M	69	Trauma	1	4	R	SP	18/20	Crystalis

**Table 2:** **Musical Questionnaire Part 1: Musical Habits.** Numbers indicate the number of choices among proposed responses and the number of positive responses (*n* = 21). Propositions for type of music were not exclusive. For the first question, the numbers indicate mean ± standard error of mean of Likert score [range]. MPIS (*n* = 6) and Crystalis XDP (*n* = 15) refer to the usual strategies used by the patients. HL: hearing loss, CI: cochlear implant.

Item		Before HL	Before CI	MPIS (*n* = 6)	CrystalisXDP (*n* = 15)
How important is music in your life?		-	-	3.7 ± 0.42	3.6 ± 0.34
Do you attend musical events?		-	-	2	9
Do you look for new musical releases?		-	-	3	2
Do you read publications on music?		-	-	2	6
How often do you listen to music?	Often	-	10	2	3
	Sometimes	-	7	4	9
	Never	-	4	0	3
How much music daily?	<30 min	5	16	2	8
	30–60 min	11	2	3	6
	1–2 H	1	2	0	0
	>2 H	3	0	1	0
	All day long	1	1	0	1

**Table 3:** **Musical Questionnaire part 2: Music Perception with Cochlear Implant.** Numbers indicate the number of choices among propositions, positive responses or Likert scores (mean ± standard error of mean, range, *n* = 21). MPIS (*n* = 6) and Crystalis XDP (*n* = 15) refer to the usual strategies used by the patients. CI: cochlear implant.

Item	Subitems/Choices	MPIS (*n* = 6)	Crystalis XDP (*n* = 15)
How does music sound with CI?	0:Unnatural-5:Natural	4.0 ± 0.26 [3,5]	2.9 ± 0.28 [1,5]
	0:Unpleasant-5:Pleasant	4.5 ± 0.22 [4,5]	3.5 ± 0.31 [1,5]
	0:Unclear-5:Clear	3.0 ± 0.4 [1,4]	2.6 ± 0.24 [1,4]
	0:Metallic-5:Not metallic	3.33 ± 0.56 [1,5]	3.1 ± 0.31 [1,5]
How do you listen to music?	As background	2	1
	Active listening	2	8
	Both	3	4
	Neither	1	0
Why do you listen to music? (answers not exclusive)	Pleasure	6	12
	Emotion	0	4
	Good mood	1	2
	Dance	3	6
	During work	2	3
	Relaxing	3	5
	Staying awake	0	1
	None of the above	0	1
When did you listen to music after CI?	Never	0	1
	<1 week after	2	4
	1–6 months	3	5
	7–12 months	1	3
	>12 months	0	2
Do you enjoy listening to solo instruments or orchestra?	Solo	1	5
	Orchestra	0	1
	Both	2	8
	None	3	1
What do you hear best or most? (answers not exclusive)	Pleasant sounds	5	10
	Rhythm	6	13
	Unpleasant sounds	1	3
	Melodies	6	12
	Voices	2	9
Can you detect wrong notes?		2	5
- detect false rhythms?		2	8
- compare performances?		5	10
- recognize a known melody?		4	14
- identify musical style?		4	11
- recognize the lyrics?		2	10
- recognize the singer?		2	9
- distinguish male/female singer?		3	13
- sing in tune?		2	3
- sing in public?		1	1
Did you train with music and CI?		5	8

**Table 4:** Music test scores as a function of musical experience and training. Total scores for music tests are presented as Mean score ± standard error of mean [range] for each subgroup.

	Total Score with MPIS		Total Score with Crystalis XDP	
	Yes	No	Yes	No
Player before CI (Yes: *n* = 7; No: *n* = 14)	24.9 ±1.34 [18.7–29.2]	25.7 ± 1.36 [15.3–32.3]	27.1 ± 1.03 [22.5–30.5]	26.7 ± 1.40 [17.8–36.3]
Singer before CI (Yes: *n* = 10; No: *n* = 11)	26.7 ± 0.92 [23–31.7]	24.2 ± 1.67 [15.3–32.3]	28.4 ± 1.14 [22.5–36.3]	25.4 ± 1.46 [17.8–31.8]
Musical training with CI (Yes: *n* = 13; No: *n* = 8)	25.5 ± 1.09 [18.7–32.3]	25.3 ± 2.03 [15.3–31.7]	26.9 ±0.90 [19–30.5]	26.8 ± 2.22 [17.8–36.3]

## Data Availability

Not applicable.
